# Re-evaluating Our Knowledge of Health System Resilience During COVID-19: Lessons From the First Two Years of the Pandemic

**DOI:** 10.34172/ijhpm.2022.6659

**Published:** 2022-12-06

**Authors:** Dell D. Saulnier, Anna Duchenko, Sierra Ottilie-Kovelman, Fabrizio Tediosi, Karl Blanchet

**Affiliations:** ^1^Department of Clinical Sciences Malmö, Lund University, Malmö, Sweden.; ^2^Department of Global Public Health, Karolinska Institutet, Stockholm, Sweden.; ^3^Swiss Tropical and Public Health Institute, Basel, Switzerland.; ^4^University of Basel, Basel, Switzerland.; ^5^Geneva Centre of Humanitarian Studies, Faculty of Medicine, University of Geneva, Geneva, Switzerland.

**Keywords:** Health System Resilience, COVID-19, Governance

## Abstract

**Background:** Health challenges like coronavirus disease 2019 (COVID-19) are becoming increasingly complex, transnational, and unpredictable. Studying health system responses to the COVID-19 pandemic is an opportunity to enhance our understanding of health system resilience and establish a clearer link between theoretical concepts and practical ideas on how to build resilience.

**Methods:** This narrative literature review aims to address four questions using a health system resilience framework: (*i*) What do we understand about the dimensions of resilience? (*ii*) What aspects of the resilience dimensions remain uncertain? (*iii*) What aspects of the resilience dimensions are missing from the COVID-19 discussions? and (*iv*) What has COVID-19 taught us about resilience that is missing from the framework? A scientific literature database search was conducted in December 2020 and in April 2022 to identify publications that discussed health system resilience in relation to COVID-19, excluding articles on psychological and other types of resilience. A total of 63 publications were included.

**Results:** There is good understanding around information sharing, flexibility and good leadership, learning, maintaining essential services, and the need for legitimate, interdependent systems. Decision-making, localized trust, influences on interdependence, and transformation remain uncertain. Vertical interdependence, monitoring risks beyond the health system, and consequences of changes on the system were not discussed. Teamwork, actor legitimacy, values, inclusivity, trans-sectoral resilience, and the role of the private sector are identified as lessons from COVID-19 that should be further explored for health system resilience.

**Conclusion:** Knowledge of health system resilience has continued to cohere following the pandemic. The eventual consequences of system changes and the resilience of subsystems are underexplored. Through governance, the concept of health system resilience can be linked to wider issues raised by the pandemic, like inclusivity. Our findings show the utility of resilience theory for strengthening health systems for crises and the benefit of continuing to refine existing resilience theory.

## Background

 The global health community has recognized the need for a systemic approach to global health challenges, and the importance of strengthening health systems along with addressing the social and economic determinants of health. Universal health coverage and drives for higher quality systems have reimagined the role of health systems in addressing the broader contextual and systemic determinants of health.^1,2^ Health systems strengthening promotes efforts that equitably and sustainably improve health services and outcomes. It acknowledges the system’s context and interactions between the system’s components and actors, not just the building blocks.^3^ As health challenges become increasingly transnational and unpredictable, health systems will need to become more resilient.

 Resilient health systems have the capacity to absorb shocks using existing resources while maintaining the same essential functions as before, adapt to them by adjusting their functions and use of resources, or fundamentally transform their functions to reduce risks in response to the shock.^4,5^ These capacities for change are governed by the rules, norms, and power structures that influence the interactions, relationships, and decisions among health system actors.^6^ Examining resilience can help build stronger health systems but will require investigating all these aspects of health systems if one wants to assess the capacities of health systems.

 Coronavirus disease 2019 (COVID-19) is defined as a complex shock, being multi-factor and multi-scale in its cause and effects. Studying health system responses to the pandemic — a shock on a global scale — provides an opportunity to refine our current understanding of health system resilience. The objective of this paper is to re-evaluate what is known about health system resilience, by evaluating the overlap in resilience concepts that are discussed in the recent literature on resilience and COVID-19 and concepts in an existing health systems resilience framework.^5^

 The widely cited Dimensions of Resilience Governance framework^5^ is used as a structure for the analysis of the literature on health systems resilience and COVID-19. The framework has four dimensions that create the capacity to absorb, adapt, or transform.^5^ Systems must be able to integrate, process, and make decisions using *knowledge* about their resources, risks, and health needs by interacting with different actors and groups inside and beyond the health system. They are able to anticipate and cope with *uncertainty *through the actions and decisions of individuals, groups, and networks in response to the shock. They must also be able to manage interactions with other systems beyond the health system (*interdependence*) and recognize the impact of contextual and external factors on the system’s behavior, capacities, and resources. Finally, systems must create a *legitimate* system that is trusted to provide socially acceptable and contextually appropriate care. The dimensions are not mutually exclusive.

 This review addresses four questions: (*i*) What do we understand about the framework’s dimensions of resilience? (*ii*) What aspects of the resilience dimensions remain uncertain? (*iii*) What aspects of the resilience dimensions are missing from the COVID-19 discussions? and (*iv*) What has COVID-19 taught us about resilience that is missing from the framework?

## Methods

 Narrative synthesis methods are well-suited to exploring the emerging literature on a new topic and synthesizing a wide range of study designs.^7^ We conducted a synthesis of data from publications discussing COVID-19 and health system resilience, and structured the synthesis around the Dimensions of Resilience Governance framework.^5^

###  Search Strategies and Selection

 A literature search in scientific databases was conducted to identify publications on health system resilience and COVID-19. The search was conducted in Medline, Web of Science, and CINAHL, using keywords on health systems, resilience, and COVID-19 (Supplementary file 1). The same literature search was conducted twice: first in December 2020 and again in April 2022, in order to identify relevant publications from all phases of the pandemic.

 Because of the scarcity of empirical research available on COVID-19 and resilience at the start of the pandemic, the inclusion criteria included any type of articles (including commentaries and opinion pieces) that explicitly discussed health system resilience in relation to COVID-19, published in English in scientific journals. We excluded articles on personal and psychological resilience at the individual level. We also excluded articles on individual or community resilience and resilience in other types of systems (eg, political systems), if they were not directly linked to a discussion on health systems. The first author screened articles by title, abstract, and full text against the eligibility criteria; articles where eligibility was unclear were checked with the remaining authors.

**Figure 1:**
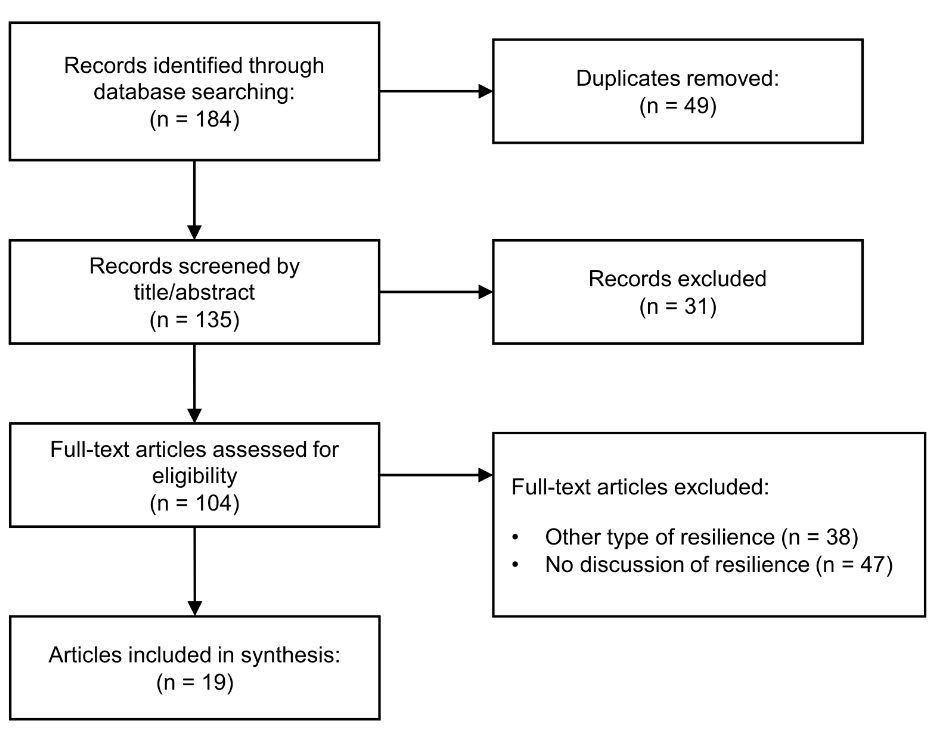
Flow Diagram of Search Results for COVID-19 and Resilience Papers in December 2020. Abbreviation: COVID-19, coronavirus disease 2019.

**Figure 2:**
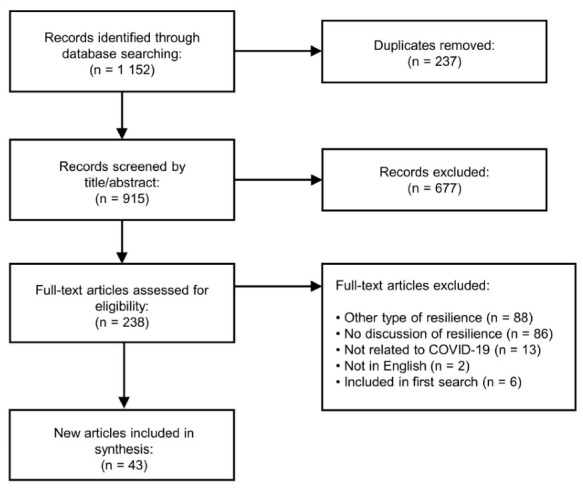
Flow Diagram of Search Results for COVID-19 and Resilience Papers in April 2022. Abbreviation: COVID-19, coronavirus disease 2019.

 The first search identified 184 articles. After screening titles and abstracts, 104 full texts were read and 19 articles were included for analysis (Figure 1). The second search identified 1152 articles, of which 238 full text articles were read and 43 articles were included for analysis (Figure 2). The main reasons for exclusion were no discussion of health system resilience or articles on another type of resilience.

###  Data Extraction and Analysis

 Any findings or discussion points on health system resilience and COVID-19 from each study were extracted onto a data form. The data were subsequently grouped by the framework’s four dimensions by the first author and reviewed with the remaining authors to confirm that the data were relevant to the dimensions it was placed under. The data were then re-assessed by the first author to determine whether there was agreement in the data about the concepts in a dimension (what was understood about the dimension) or whether there was disagreement in the data (what remains uncertain about the dimension). The authors discussed the agreements and disagreements until consensus was found. Concepts from the dimensions in the framework that were not observed in the data were noted as not discussed. Any discussion points and findings from the articles that were not described in the framework were identified and summarized. Six new thematic areas were identified during analysis that reflected these new discussion points: teamwork, health system actor legitimacy, the influence of values, equity, linking health system resilience to societal resilience, and governance of the private sector. Two analyses were conducted separately: one on the first search and another one on the second search. The results of the two analyses were compared and merged into a single analysis in June 2022.

## Results

###  COVID-19 Through the Dimensions of Resilience Governance framework

####  Which Dimensions Are Documented?

**Figure 3:**
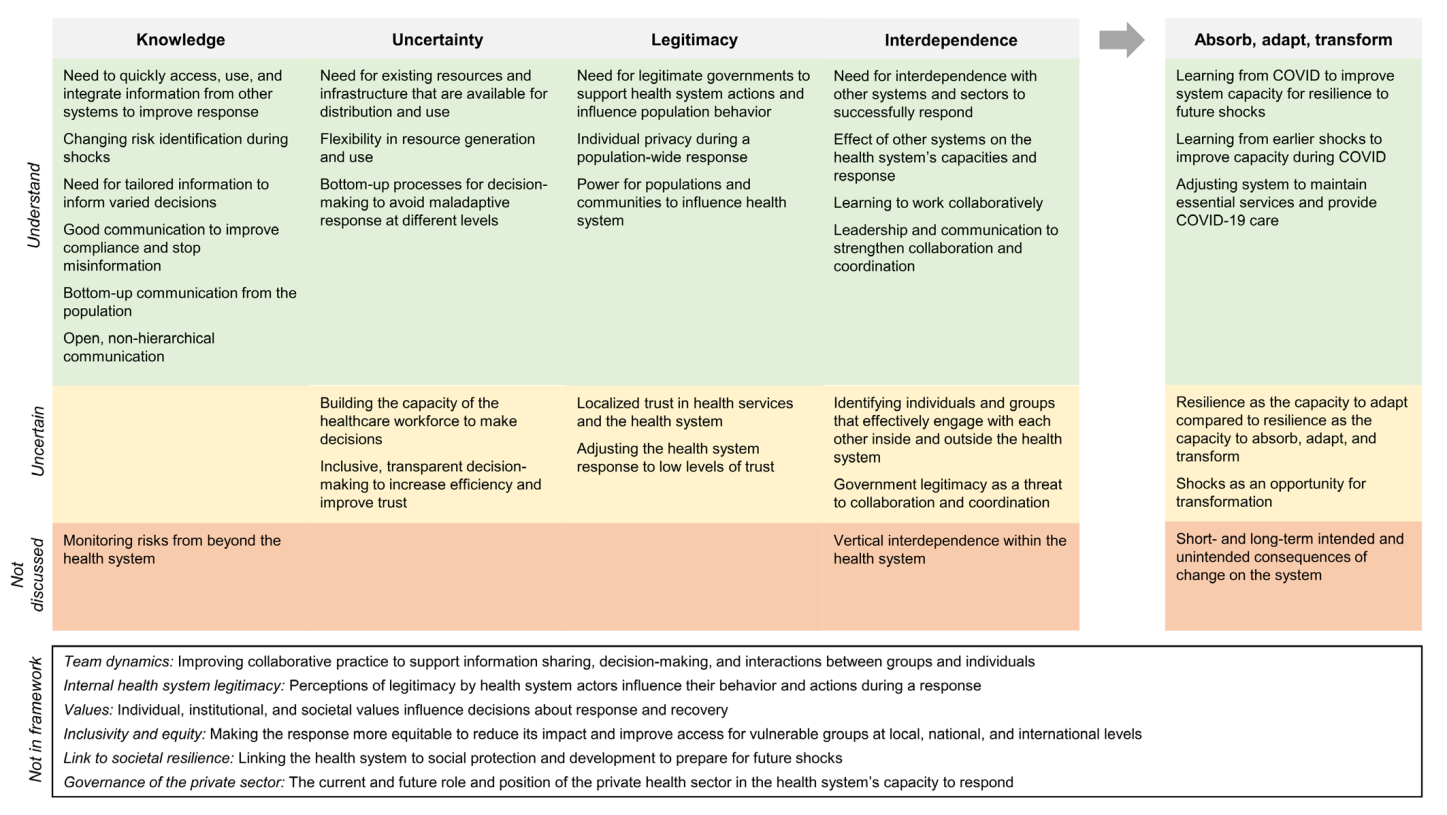
A Summary of the Main Areas Identified in the COVID-19 and Health System Resilience Literature, as They Fit Into the Dimensions of Resilience Governance Framework (Columns) and by Degree of Agreement (Rows). The white box represents areas from the literature that are outside the current framework’s scope. Abbreviation: COVID-19, coronavirus disease 2019.

*Learning*: The selected manuscripts emphasize the adaptive nature of health systems and their potential to learn (Figure 3). Health systems had to adapt multiple structures and functions to respond to the continuously changing nature of the pandemic with growing knowledge on the virus.^8-28^ Systems adapted to prevent and control transmission while trying to provide essential health services and COVID-19 services simultaneously,^13,15,17-32^ adopting strategies like implementing telehealth services and incorporating private providers into public systems responding to both needs.^16,22^

 The emphasis on learning is on individual country systems learning from previous successes or shocks to create a successful response to COVID-19^8-10,13-16,33-37^ with no clear definition of how a successful response is defined. For example, prior to COVID-19, health systems in Australia had learned from previous shocks of brush fires and a SARS (severe acute respiratory syndrome) outbreak how to mitigate widespread, system-level challenges.^38^

 Looking forward to future shocks was also recognized as an important learning process following COVID-19, to strengthen public health systems for global health emergencies.^14,23,29,34,36,38-44^ The influence of context on learning was relatively overlooked,^45^ with a few exceptions: one study describing how the 2003 SARS outbreak changed community values and the political environment for public health in Singapore and how this redefined the health system’s context prior to COVID-19,^8,10^ and one study describing how the pandemic has influenced the political climate for developing new frameworks on noncommunicable diseases in the European Commission.^42^ Several articles stated iterative and cyclical learning as a key takeaway, to continuously assess how policies and strategies performed during the pandemic over time, and to use these learnings in future policies.^23,36,42,43^

*Knowledge sharing*: Communicating, integrating, and using information from a variety of sources was regarded as important to control COVID-19, distribute resources, plan health services, and coordinate a response.^23,33-35,46-49^ The importance of knowledge to support decision-making is not surprising, as health communication, surveillance, and information gathering are the fundamentals of public health and outbreak response.^15^ Integrating knowledge from other sectors and systems into the health system can support innovation and evidence use during response, and could improve risk assessment and decision-making.^23,34,35,46-49^ However, only a few systems appeared to have integrated information from other sources, like transport and immigration data, which suggests need for improvement for future shocks. Similarly, decision-making was facilitated when standard sources of population and individual-level information, like health management and information systems, were integrated with new sources inside and outside the health system, like contact tracing apps, travel databases, and research data.^8,10,15,23,29,36,50^ Earlier outbreaks, like Ebola, have seen similar findings.^51^

 In some health systems, stakeholders changed the way that risks to the system were identified, by evolving existing information mechanisms or integrating new data sources from within and beyond the health sector.^16,24,26,27,34,44,52^ These included integrating routine administrative data to monitor health system performance,^53^ setting up informal networks among local facilities to monitor resource levels,^26^ and creating real-time algorithms for transmission risk and health service performance.^27,52^ A challenge to using information for decisions was identified when actors lacked shock-specific, service-specific, or level-specific response guidelines and information.^19,44,48,54-56^ In such situations, health system actors reported difficulty in responding without clear guidelines and protocols that were tailored to their situation.

 Consistent, clear, concise, and transparent communication from the health system and the government to the population – tailored to cultural norms and to health literacy levels – was essential to persuading people to adhere to COVID-19 regulations.^8,10,14,16,18,29-31,35,50,52,56^ For instance, when the public reacted negatively towards a tool developed to categorized the level of COVID-19 transmission risk at a subnational level in Italy, the National Public Health Institute held weekly presentations and question and answer sessions that were open to the public.^52^ The authors perceived the open and transparent sharing of information as a useful method to help quell public criticism of the tool. Top-down information sharing might combat misinformation about COVID-19 if it comes from a trusted authority, although how to prevent and reduce misinformation in the first place was an unresolved challenge.^8,31,35,54^

 Communities should be seen as integral knowledge sources in the health system, not just as beneficiaries.^23^ Individuals and communities are important sources of knowledge for COVID-19-related activities, like contact tracing, providing expertise, and sharing information on local resources.^15,26,28^ However, neglecting to involve civil society in decision-making can lead to disempowerment and distrust in the health system and government.^57^ The capacity to manage resilience may be lower in health systems that do not gather knowledge from civil society. Many governments adopted a top-down, dominant approach in the early stages of the pandemic, leaving few mechanisms to involve civil society.^23,52,58^ For resilience, it will be important to delineate which community engagement processes were successfully used during COVID-19 and which effects these processes had on the effectiveness and appropriateness of the response.

 Within the health system, open and clear communication between services, groups, and levels helped actors make timely decisions while the system adapted.^8,12,19,26,31,55,59^ For instance, a lack of information on existing supplies, health system infrastructure, and availability of resources across organizations has created challenges in distributing resources.^15^ In a study of care homes, hospice knowledge brokers, like hospice workers, were identified as a valuable resource to connect smaller facilities and less prominent actors to each other, to other local facilities, and to information on resources and guidelines.^26^ Such social networks supported actors, for example, in creating local guidelines. However, not all communication mechanisms were able to successfully integrate their target groups, who were then required to find their own ways to access information to support their decisions. An incident management system designed to streamline communication to a single point of contact was found to have excluded lower level networks and actors, reinforced top-down information sharing in the system, and duplicated work in the lower levels.^55^

*Leadership*: The role of strong and transparent leadership in managing uncertainty has been acknowledged before,^60-62^ and remains relevant for the COVID-19 pandemic. Fast, flexible, and transparent leadership for coordination and clear roles and responsibilities for individuals and groups across the system characterized effective health system responses to COVID-19.^8-10,19,30,31,48,50,56,63^ Good leadership supported and structured decision-making while the situation changed. For example, it improved communication around mistakes and was able to align similar processes between different ministries. Good leaders were also perceived as having a clear and transparent vision that let others understand their decisions. For instance, in situations with considerable unknowns, making a somewhat faulty decision quickly and swiftly was perceived by the public as a better approach than any repercussions that might have come by waiting to make a decision with more information.^48^

 To respond to the pandemic, resources and infrastructure needed to be readily available for distribution and use. Sustained, long-term investment in public health systems, particularly infrastructure and human resources, is noted as widely lacking in many systems.^13,14,17,17,18,20,23,29,63-66^ The presumed result is a system that is less flexible and less able to mobilise surge capacity.^64^ Although large amounts of financial resources have been allocated to public health and health systems for COVID-19, it has often been in the context of previous periods of austerity, underfunding, and streamlining to create more efficient medical systems. In Spain and the United Kingdom, prior reductions in spending hampered the system’s ability to scale up testing and contact tracing, and isolation and led to an undersupply of intensive care beds and ventilators.^63,64^ Chronic underinvestment in public health and primary care created a weak base leading into the pandemic,^17,18,33,67^ such as in India, where resource shortages were already an issue prior to COVID-19.^66^ Health systems with a robust and diversely skilled workforce,^17,18,23,66^ stable and protected funding,^17,20,33,65^ and reserve capacities for physical resources^17,66^ were perceived to have fewer service interruptions and to be more resilient.

 To counteract uncertainty around resource availability, systems needed to find flexible and innovative ways to generate and use resources. For funding, this included changing funding and financing rules and processes as needed (eg, removing the need to approve spending up to a certain limit), creating standardized processes to distribute funds, trying to negotiate better procurement deals, and reducing reliance on external resources.^16,19,20,28,33^ Reducing uncertainty around the availability, capability, and well-being of the health workforce meant health systems had to adapt policies to meet changing demands for services and to protect the workforce from infection and poor mental health outcomes.^8,10-12,16,19,24,26-32,34,64,68^ Adaptations varied in scale, from changing the working hour limits to changing national recruitment regulations. For example, in Trinidad and Tobago, intensive care nurses were recruited from Cuba, and in Spain, and it was allowed to hire final year medical and nursing students.^30,64^ Adapting also required organizational culture changes, such as allowing healthcare workers to work in new ways that would not have been acceptable prior to the pandemic, like exceeding maximum work hours.^26^ However, this also meant that healthcare worker occupational protections had to be simultaneously strengthened (eg, offering childcare services for healthcare workers,^27^ creating psychological support groups^16^).

 While the pandemic hit all health systems, it did not hit every level of every health system in the same way. As such, blanket policies proved to be ineffective, leading to maladaptive implementation at different levels. Using a “bottom up” and more democratic approach to include actors from different levels and sectors in the decision-making process was thought to reduce uncertainty by ensuring that decisions about the response were appropriate for differing levels of the health system.^18,19,26,54,56,69^ This was also perceived to reduce the burden on higher levels of a centralized response and to account for contextual differences in the response.^54,56^

*Interdependence*: Countries have had to conduct long-term, national level responses to COVID-19. Measures to prevent and contain COVID-19 have impacted multiple sectors. There is agreement that the breadth of COVID-19 requires better integration and awareness of interdependence during the response.^9,13-16,18,22,23,28,31,34,40,43,47,49,50,52,56,69^ First, health systems should be collaborating, coordinating, sharing information, and making decisions with civil society, academic, the private sector, and other government sectors (eg, education, labor), which required good communication skills and adept leadership at the national level. Doing so might help health systems absorb or adapt, assist in decision-making, and overcome diverse objectives across sectors.^10,20,23,30,39,49,50,54^

 Second, health systems should be strengthening internal links, such as those between healthcare facilities or between public health and clinical medicine, and links with international bodies and other health systems.^13-15,39^ Interdependence was also raised in the context of solidarity among actors to avoid competition over resources and keep the response cohesive.^12,14,59,63^ Working in a multisectoral way towards a common goal was seen as a learning process that required clear, aligned roles at all system levels, training on interdisciplinary and interprofessional values, developing cross-sectoral plans, and allowing new ways of working together to develop.^16,25,48^

 COVID-19 has forced an interdependent response at a global level. An emerging issue has been understanding how interdependence works at the international level when usual patterns of interaction have changed with the closure of borders and competition to have access to masks, oxygen, and vaccines.^16,17,20,24,36,42,46^ With COVID-19, well-established health systems in high-income countries are now experiencing different and novel problems. For instance, the United Nations International Children’s Emergency Fund initiated its first-ever emergency response in the United Kingdom to feed vulnerable children.^70,71^ Low-income countries may no longer have access to technical and financial assistance from high-income countries, who are responding to the shock themselves.^14,17,20^ Supply chains globally became unpredictable, highlighting the dominance of the international supply chain and the infeasibility of many countries to produce resources internally,^16,17,36,46^ with exceptions: interindustry technology transfers were seen in countries like Vietnam, who were able to begin manufacturing ventilators in-country.^28^ The pandemic provides an opportunity to understand interdependence at the international level and the value of effective global health governance in a world where we saw the reemergence of borders and national preference.

*Legitimacy*: Linked to the ideas on interdependence and communication, legitimacy was enhanced by having a supportive political environment that is accepted by the population as a legitimate, responsible actor in the COVID-19 response.^8,13,16,18,21,43,46,64^ Because of the scope of COVID-19, the government has been the only institution with enough legitimacy and capability to take on responsibility for coordinating and managing the response.^72^ This has created challenges for health system actors, as health systems cannot fully separate themselves from governmental legitimacy.^43^ Examples include political and social unrest, which influenced public trust in government information about the pandemic; opposition to new governmental decrees on lockdown measures; and exacerbations of prior tension between federal and regional governments.^8,13,16,17,46,64^ However, this is not new; for example, deep political mistrust has contributed to persistent transmission of Ebola in the Democratic Republic of Congo and Sierra Leone.^73,74^ With the COVID-19 pandemic, there are concerns that governments and leaders with low legitimacy will reduce the population’s acceptance of control measures (eg, reduce vaccine uptake), which would ultimately undermine the effectiveness of the health system response.^43,46^

 In conjunction with the issue of legitimate governments, individual privacy during a population-wide response appears to influence the system’s capacity to respond. For instance, contact tracing apps have been implemented in many settings, but their effectiveness depends on whether the population trusts how the data would be collected, used, and stored.^10^ In Quebec, it was observed that getting rid of a contact tracing mobile app appeared to increase public trust in the government’s response.^16^ However, social and political contexts can mediate the acceptability of control strategies; governments with top-down approaches to complying with COVID-19 regulations have been able to introduce universal, app-based technologies that curtail individuals’ movement based on contact with COVID-19 cases. The uptake of similar apps has been low in contexts that place a high value on privacy and civil liberties.^10,75^ Misinformation can also fuel mistrust in the health system,^15^ impeding the system’s ability to accurately predict and respond to changes in COVID-19 transmission and healthcare demand. Health systems that do not adequately address concerns about data sharing and misinformation may have long-term consequences on the population’s trust in the system.

 Trust is also linked to populations’ power to take ownership in the health system and influence its response. Spaces are recommended where communities and health system professionals can communicate and interact.^16,48^ At the healthcare delivery level, local facilities and actors that were able to incorporate requests from communities (eg, setting up a support hotline for full-time carers^19^) were thought to have better connections to communities and were better able to overcome policy obstacles.^19,48,76^ However, the long duration of the pandemic has required some governments to prolong unpopular and unusual interventions, like those that curtail freedom of movement, which has led to protests in multiple countries.^10,29,77^ Government responses worldwide have been publicly critiqued and politicized, in some cases reducing transparency.^15,77,78^ One recommendation includes creating permanent mechanisms for involving communities that extend beyond the shock, as long-term confidence in the government may depend on reciprocal actions by communities to adhere to health system requests and by health systems to support ownership.^79^

####  What Remains Uncertain?

 Across discussions on health system changes during COVID-19, transformative capacity has received the least attention, compared to absorptive and adaptive capacity. Examples of transformation have been described, like creating parallel systems for COVID-19 care^30^ and pushing to implement health system reforms during the pandemic.^25^ One reason for the focus on adaptation and absorption may be the tendency of the manuscripts to define resilience as the ability to prepare for and respond to shocks while maintain core functions and responding to health needs,^10,13,28-32,40,49,50,59,80^ or as the ability to withstand and adapt to a specific shock,^18,20,33,52,67,81^ which de-emphasize the capacity for profound change. The tendency to equate resilience with maintaining essential health services or with emergency preparedness may also draw attention away from possible structural and functional changes and towards short-term change in particular sub-systems or areas. This focus on performance overlooks the underlying capacities that allow a health system to fundamentally change. The absorptive and adaptive focus might also be explained by the time horizon of the articles, as about one-third of the manuscripts reviewed were written early in the pandemic.

 However, the pandemic was also reframed from a crisis to a clear opportunity to initiate reforms that could support transformation.^16,17,25,28,69^ Instead of following “the temptation to deescalate healthcare reform”^25^ during COVID-19, the pandemic was a chance to move away from the status quo. For instance, the pandemic changed the perceptions of recent public management approaches in Quebec and created new possibilities to change health system management structures.^16^ Reconciling the predominant focus on absorption and adaptation and the view of crises as a chance for transformation will require recognizing that endless absorptive and adaptive capacity are unsustainable since health systems are embedded in an unpredictable, constantly changing context.^82^

 Although the necessity to integrate and use multiple forms and sources of information is clear, it is still unclear which sources and forms are most influential on the system’s ability to respond and its capacity for resilience. While connecting knowledge from sources like communities and other sectors to the response has clearly been described as beneficial, knowledge goes beyond linking sources together. If the process of producing, collecting, integrating, and interpreting knowledge can be strengthened, it may prove to be protective to the health system’s response to future shocks.^42,48^ For instance, investing in local research capacity to generate localized knowledge, such as local vaccine production, may reduce a system’s reliance on the international community during future shocks.^34^

 An emerging idea from COVID-19 is the need to build the capacity of the healthcare workforce and other health system actors to make decisions in response to a shock. This moves beyond what decisions can be made and by whom, to how to build individual and group capacities to act.^19,26,28^ For example, care home healthcare workers improved their confidence in their leadership skills when the unique skills that they developed during the pandemic, such as learning how to negotiate with local suppliers, were recognized and repeatedly used.^50^ Supporting the capacity of health system actors to make and act on decisions may be useful for their ability to adapt to change in the future.

 A second emerging area is the need to make the decision-making process transparent, including stating a clear rationale for decisions. The different decision-making processes for COVID-19 responses has been relatively overlooked; this may be because many national-level response decisions were considered to be political decisions that went beyond a public health purpose.^83^ Policy makers have been under pressure to quickly assess a changing situation, react to new information, and provide expertise on a novel virus.^84^ Several authors argue that clarifying the process would have reduced the uncertainty among health system actors that the decisions were correct and could be trusted.^23,38,44,48,52^ Governments were criticized for not openly discussing the uncertainty of the decisions they were making and that decisions could change in future circumstances.^23^ However, distrust in decisions may relate more to a described lack of inclusion in the decision-making process for groups of health system actors.^48,49,55,56^ An exclusive decision-making process may have led to inefficiency in health system responses. Without access to a centralized incident management system that made decisions about care, primary healthcare actors had to spend additional time advocating for their needs and decided to duplicate decision-making streams.^55^ To understand the dimension of uncertainty, future analyses will need to address how the process of decision-making influences the system’s ability to act, including the effect of social relationships.

 Many COVID-19 public health interventions have relied on individuals and local communities to change their behavior and comply with regulations. The willingness of the general public to comply is closely tied to trust in the government, the health system, and the acceptability of the interventions. There is still uncertainty on how to understand localized trust and how to harness it to improve compliance to national policies requiring rapid collective behavior change. Trust in public health messaging and the effectiveness of local responses might be improved by involving and soliciting feedback from community members and local influencers.^15,30,44^ In theory, trust might be stronger in rural areas and with the primary care sector, where there are closer links between service providers and communities.^12,36^ Future discussions on resilience will need to explore the implications of implementing top-down interventions on legitimacy.

 Health systems may need to adjust to low levels of trust while responding to COVID-19 and other crises, although the most effective and appropriate ways to do so remain unclear.^28,49,52,67^ For example, maximizing vaccination uptake and coverage is essential to preventing transmission but is highly dependent on the population’s trust in vaccinations and the health system. In some contexts, it may be possible to implement policies that mandate vaccination, like previous COVID-19 policies that have emphasized personal responsibility.^10^ In others, this may be legally or contextually impossible. How should health systems adjust if they are unable to meet vaccination targets? Some given strategies to adjust include reorienting health system actions to the population’s wants, rather than the system’s preferences (eg, expanding routine vaccination delivery systems to locations that are more convenient to patients^67^) and incorporating quality of care domains into all aspects of emergency preparedness and response.^49^ It is likely that in future crises, health systems will need to rethink their response strategies to create socially acceptable solutions in light of the population’s level of trust.

 The ability to lead a robust national response to COVID-19 requires governments to work across sectors and systems. There remains relatively little describing which individuals and groups are involved in effectively engaging with each other, both inside and outside the health system. Making use of the individuals and groups who are able to efficiently interact with others is likely to be supportive of the health system’s capacity to change and respond to a shock.^44,55^ For instance, one individual primary care stakeholder was able to access information and decision-making processes and then informally link them to other primary care stakeholders.^55^ Such individuals may be the key to integrating health system groups and services during shocks, which can support their response to shocks.^85^ However, in some contexts, political tensions between groups and organizations appear to be a threat to the ability of groups to interact and the health system to respond. The political independence of pandemic agencies has been called for in the United States, to protect them from political threats,^46^ and growing sectarianism amid political turmoil in Lebanon was thought to have weakened the health system’s mechanisms to collaborate and coordinate.^17^ The types of individuals and groups that are effective at engaging with others will likely differ by context and health system structure, but identifying the underlying factors to their success could be worthwhile exercise with practical implications for future responses.^86^

####  What Resilience Aspects Are Missing From the Discussions?

 Health systems have needed to react quickly and constantly to COVID-19. Certain short-term consequences from the changes are predicted, like a backlog of unmet health needs that will have to be addressed following the cancellation of routine health services. The longer-term consequences on the system and the context have been harder to observe. For example, changes in service utilization patterns have impacted the income of providers who use volume-based payment plans.^20^ A challenge with the pandemic has been the need to act quickly to a rapidly changing situation, making outcomes difficult to predict and little time to assess possible consequences. One consequence of rapid action was observed in a study of care homes, where the homes were forced into a new, dependent relationship with government supply chains, following a government decision to reorganize the supply chain to prioritize hospitals; the consequence was a slower, more expensive, and inefficient supply chain for the care homes.^26^

 Health systems need to be able to monitor risks as they develop beyond the health system.^5^ The pandemic has created a number of risks to the health system besides the virus itself, through the response to the pandemic and its impact on societal systems. COVID-19 has been a prime example of these risks. The pandemic’s impact on economic and food systems led to growing malnutrition, with estimates that an additional 6.7 million children under five years old would become wasted and an additional 128 000 child deaths from malnutrition would occur in 2020.^87^ At the same time, essential nutritional services like community screening and vitamin supplementation have been disrupted or are no longer accessible in vulnerable countries.^88^ The pandemic has also threatened health system resources, such as reduced financing because of economic recession.^20^ Although these risks were acknowledged in the articles, there was little discussion on how they could be monitored or identified.

 The necessity of cross-sectoral and national and international-level interdependence for resilience is well-described in relation to the pandemic; the vertical interdependence of the health system is not. Health systems are interdependent across scales and subsystems as well as with other systems, which is fundamentally reflected in our results on knowledge sharing and uncertainty. The link between effective teamwork and health system actor legitimacy and vertical interdependence is also acknowledged (further discussed in the section “What is missing from the framework?”). Yet the causes of failures at different health system scales or subsystems or ways to strengthen vertical interactions have not been explored.

####  What Is Missing From the Framework?

 We identified six main areas omitted in the resilience framework that were essential elements during the pandemic: teamwork, actor legitimacy, influence of values, explicit consideration of equity and fairness, linking health system resilience to societal resilience, and governance of the private sector.

*Teamwork:* The pandemic shows that information flows, decision-making, and interactions between the systems levels are impacted by the quality of teamwork among health system actors and groups.^16,18,24,26,55,56,65,69,76^ The ability for actors and healthcare workers to successfully work together as colleagues and in teams relies on trust, power dynamics, and perceived inclusion in groups and in processes. Multi-country evidence shows that the resilience of the health workforce requires more than funding, governance, and good organization of services^24^: since teams constitute a sub-system of the health systems, resilience is not possible without resilient teams with supportive team dynamics.^18,65^ Power imbalances among groups and team members and lack of trust-building mechanisms like whistle-blowing or feedback tools led to uneven levels of trust between healthcare providers themselves and with other actors.^16^ The lack of such mechanisms was especially felt with top-down approaches, which appeared to decrease the workforce’s collective belief in the capacity of local and national governments.^16,26,76^ ‘Low power’ actors in the health workforce, like primary care and care home staff, may regularly experience isolation from operations and decision making, not only during shocks. The lack of access to information and support created an environment of isolation and little solidarity among actors.^26,55^ An expected consequence to low trust and solidarity among teams is an unwillingness for various actors to cooperate, share information, and work together.^26,69^ Training in collaborative practice and interprofessional teamwork, including evidence-based dissemination of information and enforcement, addressing data ownership and confidentiality, may be key to address the issue.^56,65^

*Health system actor legitimacy*: In addition to community legitimacy, actors in the system must also perceive the system as legitimate. Their perceptions of the governance mechanisms of the health system will influence their behavior in carrying out and committing to health system actions (eg, following guidelines).^16,18,26,41,47,55,69,76^ Legitimacy extends to both the governmental services as well as the non-governmental sectors and actors, such as professional unions who protect healthcare worker rights, non-governmental organizations, and the private sector. Groups that were often excluded from decision-making or information flows, like community healthcare workers, nursing home personnel, primary care providers, and the public health workforce, had to adapt on their own. This led to resentment, decreased trust, and decreased willingness to cooperate with local governments and commit to centralized strategies, particularly in the groups that lacked mechanisms to disagree with more powerful actors.^16,26,47,76^ Other existing issues, like hostile work environments for healthcare workers, existed before the pandemic but exacerbated the workforce’s mistrust in local and central governments and their frustration during the crisis.^55,69^ Not only is remuneration for the health workforce important, but also respect that is embedded in operations, communication, and practices.^41^

*Values*: Individual, institutional, and societal values have shaped many of the decisions that were made in responding in COVID-19 and the objectives of the response and recovery.^16,19,35,48,67,69,76,79,89^ With the pandemic presenting a situation of great uncertainty, decisions often had unknown outcomes and consequences when they were made, leading to ethical dilemmas among decision makers.^48,79^ For example, guidelines and ethical frameworks could be lacking or conflict with the context, the available resources, or community, group or individual values – all of which constituted a major barrier to providing services and implementing new decisions.^16,76,79^ The act of prioritizing services for different groups can be a value-based judgement, as well as a technical decision. Priority setting both in evidence generation and resource allocation were important not only during the shock: subsystems and areas that were neglected pre-shock appeared as weak links in the response, creating a risk for next crisis.^69,79^ It is likely that individual, institutional, and societal values have impacted health system responses in other ways, since values will shape the way that the dimensions of the framework manifest in a health system.

*Equity*: Fostering an equitable, inclusive response to COVID-19 at local, national, and international levels has been crucial to containing the pandemic (although sometimes failing), improving access to and quality of services for vulnerable groups, and reducing the health impacts on populations.^9-12,14,20,40,58,65,65,69,90,91^ Focusing on vulnerable groups, like migrant workers living in densely populated housing, can help prevent transmission.^10^ The pandemic has shown system weaknesses and existing disparities in accessing and receiving care along social and economic lines, in contradiction to the principles of universal health coverage.^92-94^ At the local and national level, disadvantaged and vulnerable groups, such as the homeless, people living with chronic illness(es), and those unable to afford healthcare, have been disproportionately affected by COVID-19 and the indirect effects of COVID-19-related interventions like lockdowns.^14,20,94^ Lockdowns and other restrictions have disrupted, sometimes purposely, access to and provision of reproductive and sexual health services, including support for gender-based violence, contraceptives for teenagers, and abortions.^58^ Uptake of adaptive measures is also greatly influenced by social inequalities. For example, Colombia introduced telemedicine services but struggled to reach the one-third of the population that did not have access to broadband.^69^ Internationally, existing resource inequalities between higher and lower-income countries are likely to be worsened by COVID-19,^95^ and lower-income health systems may have greater difficulty providing health services and accessing and distributing resources like vaccines.^96^ To address inequities, resilient health systems should be able to identify vulnerable groups in the population, detect processes and services that sustain inequity in the system, and adjust in response. They must also recognize that health systems can also generate more inequity through their own actions, such as not being proactive at targeting minorities or ignoring the intersectionality between areas such as gender inequality and climate, and find ways to address the root causes of inequality that affect health outcomes and the system’s performance.^90^

 Equity is linked to all the dimensions in the resilience framework. Similar to earlier outbreaks,^97^ trust is affected by the ability of individuals and groups to access, pay for, and receive quality care when systems are primarily focused on COVID-19 care^9,14,58^; knowledge sharing can help to identify vulnerable groups,^15^ and so on. COVID-19 has drawn attention to how health systems may only be able to manage resilience at one level and for particular groups, perpetuating equity gaps. Health systems may be capable of managing resilience in urban areas, for example, but unable to absorb, adapt, or transform in rural areas that can be under-resourced and serving a more disadvantaged population.^12^ This raises questions around the comparative value of the dimensions and interactions within them at different levels, and if they are weighted equally in their contribution to resilience capacity.

*Transsectoral*: The COVID-19 pandemic has been called a syndemic, an interaction between the biological threat of COVID-19 and social, economic, and environmental factors that results in adverse outcomes.^98^ Factors like poverty and social and gender inequality have created the inequity and disporportionate impact of COVID-19 on vulnerable and disadvantaged groups. While universal health coverage is key to ensuring that all people are able to obtain good quality health services without experiencing financial hardship as a result, the responsibility for social care and protection are outside the remit of the health system. Yet these factors are a large contributor to the outcomes that the health system is responsible for.^1,34,99,100^

*Private sector*: For the public and private sector to respond in a complementary manner, strong governance of the private sector is required.^58,64^ During the COVID-19 pandemic, private sectors played a major role in the ability of multiple health systems to coordinate a response and to maintain routine health. They also affected the amount of financial resources available to the public sector and the speed with which the public health system was able to react to the pandemic.^10,12,13,30,39,59,64^ However, it is the public sector that runs the COVID-19 response in most countries.^29^ Some public sectors have been weakened by reduced investment in state-funded, government-run systems, leading to poorly integrated services, less trust in the health system as a whole, and poorer coordination between sectors during the response.^13,29,39,58^ For example, in India, the private sector was able to fill gaps in lab testing and COVID-19 care when the public sector was unable to accommodate additional patients. The need to use private providers resulted in disproportionate economic effects on lower socioeconomic classes, with knock-on consequences to health outcomes and trust in the system.^13,58^ In some cases, robust oversight of the private sector, like introducing centralized purchasing, helped to reduce competition over system resources, reduce profiteering, and strengthen collaboration between the private and public sectors. Interactions can be structured to promote resource sharing and collaboration but it requires governments to have the necessary regulatory capacity to ensure that public-private approaches are cost-effective, accountable and transparent.^10,20,28,30,36,47,49,59,64^

 How should the private sector be governed and regulated to promote resilience? In general, the role and position of the private health sector in the system’s overall capacity to respond to current and future shocks has not been well-studied. The resilience framework makes no distinction between governance of the private and public sector, under the assumption that the four dimensions are relevant to resilience capacity for the whole system. However, like with equity, resilience capacities may vary greatly between the public and private sectors, particularly in systems with highly unregulated private sectors. In turn, this likely influences the four dimensions and the system’s capacity to manage resilience. Differentiating between the governance of health system levels and sectors could help further explain resilience capacity.

 Table shows summary of 63 articles included in the review.

**Table:** Summary of Articles on COVID-19 and Health System Resilience

**First Author **	**Date Published**	**Objective or Purpose**	**Main Findings**
Legido-Quigley^64^	March 2020	To describe the resilience of the Spanish health system during COVID-19 using the health system building blocks.	Long-term underinvestment had weakened health services, and financial resources were required to support regional responses. Coordination across levels and public compliance has been good but must be reinforced over time.
Gopichandran^29^	April 2020	To discuss the components for building health system resilience in India, based on the response to COVID-19.	Health governance and law must be systemically strengthened after underinvestment in public health and the public system to promote transparent communication. Disease surveillance and laboratory systems must be better coordinated and more robust. Clinical and public health human resources need to be developed.
Hsieh^5o^	April 2020	To describe the resilience of the Taiwanese health system during COVID-19.	Strong coordination between private and public sectors, good oversight, and clear, transparent communication aided in the response and in creating trust. Information was shared across sectors. Policies to promote community resilience were put in place.
Kirchhof^63^	May 2020	To compare the response to the COVID-19 pandemic in the United Kingdom and in Germany.	Centralized decision-making slowed the response and reduced flexibility. Localized decision-making increased innovation, speed, communication, and learning. Long-term underinvestment had weakened both systems.
Legido-Quigley^8^	May 2020	To outline core dimensions of three resilient health systems in Hong Kong, Singapore, and Japan during COVID-19.	Integrating services in the health system and across other sectors improved the systems’ ability to absorb and adapt to COVID-19. Trust in the health system and government was highly important but harmed by fake news and misinformation.
Peiffer-Smadja^31^	June 2020	To describe the key elements of a French hospital’s COVID-19–response.	The hospital needed to address the indirect effects of the outbreak on all departments, ensure committed and effective leadership, support healthcare workers, and organize communication with the public.
Hunte^30^	June 2020	To describe the resilience and responsiveness of Trinidad and Tobago’s health system during its response to COVID-19.	The system’s response was built on evidence-informed policy and interdependence. Governance, coordination, informed decision-making, and leadership were critical to the system’s resilience.
Garcia Elorrio^68^	June 2020	To discuss strategies for PHEwith focus on redesigning and strengthening health systems to make them more resilient.	The current COVID-19 pandemic presents an opportunity to adopt a comprehensive approach to quality of care that integrates quality planning, quality control and quality assurance with improvement science to achieve sustainable, large-scale adoption. As health systems start or continue to adopt these methods, it is important to assess their effectiveness rigorously, accompanied by proper management, accountability and governance systems and structures.
Dasgupta^58^	July 2020	To explore the impact of COVID-19 on sexual and reproductive health and rights through an intersectoral lens.	Restrictive policies, disruption of public health services for sexual and reproductive health, and reliance on private sector services have eroded trust and reduced community resilience.
De Ceukelaire^39^	July 2020	To learn from country responses to COVID-19 so far.	Integrated services and trust in the health system and government appear beneficial for resilience. Underinvestment in public health systems and increased privatization have hampered coordination, service delivery, and confidence in the system.
Sheehan^9^	July 2020	To identify lessons from COVID-19 to improve public health system preparedness for climate-related emergencies.	Cross-sectoral responsibilities need to be clearly delineated. Health information systems need to be responsive, targeted, and updated frequently. Enhancing community engagement can benefit preparedness. Health needs to be linked to economic and societal development. Leadership requires adaptive capacity and integration into planning.
Mazingi^32^	August 2020	To explore vulnerable points in surgical systems from past outbreaks and COVID-19.	Surgical infrastructure, the workforce, and service delivery are most vulnerable to shocks. Recommendations to improve resilience include improving telemedicine, maintaining surgical services through risk-based approaches to delivering services, and improving policies to support and protect the workforce.
O’Sullivan^12^	August 2020	To provide a commentary on the rural primary healthcare sector during the COVID-19 pandemic.	Pandemic policies and communication should be tailored to address rural risks and contexts. Primary healthcare is a staple of rural care and has benefited the response through adaptiveness, flexibility, local decision-making, resource management, and regional collaboration. COVID-19–related responses like telehealth may strengthen rural care post-pandemic.
Gupta^13^	August 2020	To better understand the extent of resilience in India’s health system.	Chronic underinvestment in public health and the public health sector have weakened the health system, hampered preparedness, and reduced trust and confidence in the system.
Etienne^40^	August 2020	To identify transformative changes in current approaches to health systems and development using lessons from COVID-19.	Health needs to be linked to economic and social development, followed by integrated policy and planning. Health systems should focus on equitable access to health services like primary care, and public health functions should be strengthened.
Costa Font^59^	August 2020	To determine which characteristics of managed competition can make a difference in the management of a pandemic.	Regions in Italy that adopted a centralized model of managed competition appeared more resilient, through better and faster coordination, cooperation, and information sharing.
Meyer^11^	September 2020	To illustrate the importance of investments like telehealth for rural healthcare to support resilience during COVID-19.	Policy and services were quickly adapted during COVID-19 to enable telehealth care, but policy reforms are needed to address persistent challenges like access and affordability of care.
Chua^10^	September 2020	To present Singapore’s COVID-19 response using two health system resilience theories.	Clear leadership and governance allowed flexibility, maintaining health services. The government communicated quickly and transparently, made crisis financing available, and had a legal foundation to implement policies. Issues of trust and inclusiveness remain.
Collins^14^	October 2020	To highlight the overarching areas that need to be prioritized to enhance governments’ ability for effective prevention, alert, and response to emergencies to improve the health of their populations, so they become more resilient to health shocks.	Public health systems and public health capacities need to be strengthened and require greater investment. International and multisectoral coordination and solidarity could have improved preparedness and response. Health inequities should be reduced to prevent negative outcomes and improve community resilience. Sustained and appropriate communication can support community resilience and empowerment.
Lal^15^	October 2020	To assess lessons learned from deploying health information systems during COVID-19 and Ebola outbreaks to optimize preparedness and response actions.	Governance and coordination should be strengthened and aligned to global health agendas. Health systems infrastructure and resources should be built up and integrated into primary care. Community engagement can help improve information accuracy and trust and prevent misinformation.
Cuschieri^27^	November 2020	To summarize pandemic preparedness measures in Malta and the impact on routine services.	Malta and its sole acute hospital coped well with the first wave of COVID-19. The increased capacity will serve well for the anticipated combined annual influenza and the second wave.
Haldane^90^	December 2020	To argue for transformative resilience following COVID-19.	Health systems need to recognize their role in human systems and respond to crises in ways that protect the health system and other systems from harm in the future.
Lal^89^	December 2020	To understand how health systems with strong GHS and UHC policies fared during COVID-19.	Integrating GHS and UHC can improve interconnectivity between health system levels and actors. Broader funding pathways can help unify health systems. Joint health and development agendas can support resilience. Power dynamics should be included in resilience assessments.
Daszak^46^	February 2021	To review the US approach to pandemic preparedness and its impact on the response to COVID-19.	Authors identify six steps that should be taken to strengthen US pandemic resilience, strengthen and modernize the US healthcare system, regain public confidence in government leadership in public health, and restore US engagement and leadership in global partnerships to address future pandemic threats domestically and around the world.
Marshall^26^	February 2021	To examine care home management responses to COVID-19, considering their position in the health system.	Centrally organized responses led to resource constraints, additional work, and a lowered ability to make localized decisions in care homes. Resilience was a result of the ability of staff and teams to network to get around challenges brought by the centralized response.
Ahanhanzo^34^	March 2021	To explore if African countries have leveraged experiences from past epidemics to build resilience and response strategies.	West African countries continue to build resilience and responsiveness into the health systems. Coordination mechanisms have also improved across the region. There are gaps that might lead to overdependence on the global community to meet local needs.
Narwal^66^	March 2021	To derive lessons on resilience from patient safety issues during COVID-19 in India.	Inadequate resources and infrastructure from chronic underinvestment in public health systems, a lack of reliable data, and limited leadership and regulation impacted patient safety. Greater investment in the public sector may minimize future risks.
Hamadeh^17^	April 2021	To describe the current resilience mechanisms of the Lebanese public primary healthcare system, developed from earlier shocks.	The primary care system can work and share information collaboratively across sectarian lines and with multiple actors, and to work effectively with all population groups.
Pilevari^43^	April 2021	To describe the Iranian health system, with an emphasis on how resilience plays a role with the stressor of COVID-19.	Inefficiencies in the health system were detailed, with recommendations for equitable health financing, proper training of personnel and use of facilities, and strengthening intersectoral cooperation.
Romani^44^	April 2021	To compare and contrast the resilience of two different health systems in Italy during COVID-19.	The health system of one province appeared to have more resilience capacity, largely due to strengthening resources where strain was felt, diverting resources where feasible, and relying on a COVID-19 case dashboard to inform risk exposure levels continuously.
Alami^16^	June 2021	To assess systems preparedness and resilience towards emerging infectious diseases based on Palagyi framework.	Resilience and the ability to adequately respond to a systemic and global crisis depend upon preexisting system-level characteristics and capacities at both the provincial and federal governance levels.
Gebremeskel^47^	June 2021	To explore the role of community health initiatives in health system resilience in African countries.	Priority should be given to community-led health initiatives and health workers; ensuring reliable medical and diagnostic supplies; fostering evidence-based practice; and raising additional revenue to boost health system financing within the region, to boost health system resilience at the community level. All require stronger health system governance, including multisectoral collaboration within countries and regional collaborations.
Hasan^18^	June 2021	To synthesize the evidence on integrated health service delivery during COVID-19 in LMICs.	Organizational integration across health system levels and building blocks can strengthen intersectoral coordination. Alternative service delivery mechanisms can support innovation to ensure uninterrupted routine care services.
Bashier^41^	June 2021	To present opinions and expectations about the anticipated changes in the future of public health at the global, regional, and national levels, based on a need for better governance and stronger and more resilient health systems.	Coordination and collaboration among countries and stakeholders in different multilateral and global initiatives, as well as of evidence-based solutions and a strengthened health workforce, are critical to resilience. Agencies, systems, and individuals must be resilient enough to cope with any expected changes.
Haldane^28^	June 2021	To review COVID-19 responses in 28 countries using a health systems resilience framework.	Resilient health systems were considered to have comprehensive responses that integrated health with social and economic considerations, adaptive capacity within and beyond the health system to meet the needs of communities, preserved functions and resources within and beyond the health system to maintain pandemic-related and other care, reduced vulnerability to catastrophic losses in communities, and continually learned and adjusted.
Kwon^20^	July 2021	To provide approaches to strengthen health financing and resilience, based on the impact of COVID-19 in Asia and the Pacific.	Sufficient resources, fast and flexible funding mechanisms, multisectoral cooperation, and adequate public health funding supports the system’s ability to prepare for and respond to health emergencies. Innovative financing can mitigate risks.
Smaggus^38^	July 2021	To investigate how government actions in New South Wales, Australia and Ontario, Canada related to health systems resilience.	Both governments focused on reactive resilience, while fostering proactive resilience could be more beneficial to society. Having a proactive vision of resilience and acknowledging the complexity of health systems could improve resilient performance in healthcare.
Phillips^48^	August 2021	To explore information asymmetries during COVID-19 and how they pertained to the UK government’s decision-making.	In times of uncertainty, actors of different levels and sectors looked to acquire new information to minimize errors in decision-making. Most innovation occurred in management and organization, rather than radical innovations.
Fiske^79^	August 2021	To discuss critical ethical concerns in COVID-19 pandemic response, as they relate to building resilient health systems.	Healthcare system resiliency hinges on ‘ethics by design’. Key ethical concerns are the distribution of scarce resources; research ethics; structural inequities; and solidarity and social cohesion. Health systems need to proactively integrate ethical considerations into their design and operation, and ethicists need to be a part of pandemic response.
Amul^35^	August 2021	To discuss key policy responses in Southeast Asian countries’ approaches to COVID-19, including resilience.	Countries with existing resources, infrastructure, and future resilience plans were at an advantage during COVID-19. Workforce and multisectoral cooperation were critical to the most successful responses.
Balqis-Ali^56^	August 2021	To explore public perspectives on health systems’ response towards COVID-19, and to identify gaps for health systems strengthening by leveraging on WHO health systems’ building blocks.	Since all building blocks influenced service delivery during COVID-19, with governance having a cross-cutting effect on the response, addressing macro-level problems with short and long-term strategies may help support resilience capacity.
Hodgins^19^	September 2021	To assess innovations and changes created during COVID-19 in children’s health services in Australia.	Health systems values of equity, integration, and support of the workforce shaped the response. Non-hierarchical governance structures, responsiveness, and a clear vision supported innovation and change.
Burke^25^	September 2021	To examines whether and how the Irish government’s pandemic response contributed to health system reform and increased resilience.	Crisis can open a window of opportunity for transformative changes, which can be utilized to initiate or continue reform implementation, as was the case of Irish Slaintecare reform during the COVID-19 pandemic.
Plagg^91^	September 2021	To illustrate the regional differences in response to and outcomes of COVID-19 in Lombardy and Veneto, Italy.	Flexible health services allowed for the greatest success in containing exposure and mitigating risk, as well as having a strong primary healthcare sector with good coordination of care between primary and secondary/tertiary levels of care.
Sundararaman^36^	September 2021	To reflect on what makes for resilient and prepared health systems.	Five design features—organization of primary healthcare services, planned surge capacity, robust surveillance integrated with health management information system, ability to scale-up production domestically, and a government that recognizes the importance of health systems that are adaptive—may create resilience capacity.
Khalili^65^	October 2021	To identify directions to address and support interprofessional resilience at all levels of healthcare.	Team resilience is critical to organization-level and system resilience, by optimizing collaboration and information sharing, and supporting the well-being of the workforce.
Larson^67^	October 2021	To describe lessons learned from COVID-19 about strengthening vaccination programs.	Routine vaccination should be prioritized as an essential health service. Access can be expanded by providing services through non-traditional vaccinators and alternative sites. Strengthened data systems can improve program performance. Building trust and confidence can improve uptake and reduce misinformation.
Tokalić^81^	October 2021	To examine how Croatia and Bosnia and Herzegovina dealt with COVID-19 in terms of health systems resilience, following their recent wars and natural disasters.	Croatia and Bosnia and Herzegovina had health systems that learned from and adapted to previous shocks of war and natural disasters. Strategies included an integrative homeland security system, plans on how to mobilize healthcare workers as needed, and learning how best to improvise in circumstances when there is a lack of resources.
Martin^76^	November 2021	To explore the influence of health system governance on community care staff during COVID-19 in England.	The central control over resources and priorities led to limited control over resources, limits on decision-making, and a lack of a voice for community staff transitioning to the crisis. Collective belief in individual and organizational capacities may support transitions.
Leslie^55^	November 2021	To examine competing resilience-focused responses to COVID-19 in Canada.	Stakeholders had competing visions on how to achieve resilience. Integrating and including all stakeholders in centralized planning should be a priority.
Orhan^42^	November 2021	To examine how to reduce the impact of NCDson health systems in the European Union in light of COVID-19.	COVID-19 has created momentum to develop policies to build resilience towards NCDs. National-level policies had weaknesses in protecting the Union from cross-border threats. Multi- and cross-sectoral collaboration is expected to lead to resilience.
Riccardo^52^	November 2021	To examine a COVID-19-monitoring tool implemented in Italy and its direct and indirect effects.	While the tool was able to detect cases and increase precautions quickly to mitigate COVID-19 exposure, it faced public criticism for being too sensitive. Public forum question-and-answer sessions held by the government helped reduce criticism.
Aristei^54^	January 2022	To overview the policies, regulatory frameworks, and legislation on health emergency management at the European and global level.	Crises highlight existing gaps in cooperation and collaboration within both horizontal and vertical levels, as well as regulatory conflicts.
Singh^23^	January 2022	To catalog the responses of Member States of the WHO Regional Office for South-East Asia on lessons learnt throughout the pandemic.	16 topic areas were identified as the most important lessons learnt during the pandemic. The importance of long-term oriented thinking on behalf of policy makers was highlighted as fundamental to strong health systems.
Thomson^33^	January 2022	To explore the resilience of health financing policy to economic shocks by reviewing policy responses to the 2008 financial crisis and COVID-19 in Europe.	Countries with social health insurance schemes fared worse in both the 2008 financial crisis and COVID-19 in health systems management and financing. Health financing sources should be diverse, health sector funding should increase, and systems should learn from previous health financing shocks.
Karamagi^80^	February 2022	To develop methods to measure inherent and targeted resilience, using data from 47 African countries.	Both capacities are necessary to address predictable and unpredictable shocks. Inherent resilience was low across countries, with transformative capacity least developed.
Øyri^21^	February 2022	To analyze the situated, structural, and systemic resilience Norway’s health system had in its COVID-19 response.	While the Norwegian government faced criticism for relatively modest COVID-19 precautions, it had adaptive ability to shift resources within health systems.
Mustafa^49^	February 2022	To assess the extent that preparedness plans integrate essential health service continuity.	Emergency plans lacked local stakeholder engagement in planning, and few plans included maintenance of essential health services. Plans lacked quality of care considerations and plans to monitor capacity for essential service functionality.
Shaw^22^	February 2022	To describe the resilience of a newly developed digital-health tool implemented during COVID-19.	Cohesiveness supports a strong response to large-scale threat like COVID-19. The ability to adapt depends on intersectoral collaboration and tolerated innovation.
Arsenault^53^	March 2022	To assess the immediate effect of the pandemic on 31 health services in 10 countries.	Many health systems failed in regard to capacity to absorb stress and perform at the same level, and being responsive to preexistent needs. Countries must strategically address this incapacity for future emergencies.
Burau^24^	March 2022	To analyze the adaptive, absorptive and transformative capacities of the health workforce during the first wave of the COVID-19 pandemic in Europe and to assess how health systems prerequisites influence these capacities.	Regardless of health system prerequisites, the health workforce in different countries was able to actualize absorptive, adaptive and transformative capacities in COVID-19 pandemics.
Turner^69^	March 2022	To examine how responding to COVID-19 has influenced personal and organizational resilience among health system leaders.	COVID-19 spurred changes within the health sector and had unexpected and positive and negative effects on adaptive capacity at different levels.

Abbreviations: COVID-19, coronavirus disease 2019; WHO, World Health Organization; PHE, Public health emergencies; GHS, Global health security; UHC, Universal health coverage; NCDs, Noncommunicable diseases; LMICs, Low- and middle-income countries.

## Discussion and Conclusion

 Our narrative review of health systems resilience and COVID-19 shows a moderate level of agreement and understanding on the concepts of health system resilience from the Dimensions of Resilience Governance framework. We have identified lessons from the discussions on COVID-19 that show the limits of the framework and point to a wider role for resilient health systems in protecting health and in society. However, there are several concepts from the framework that do not appear in the COVID-19 literature.

 As complex systems, the changes made during COVID-19 will have intended and unintended consequences for the health system.^101^ Changes that were made to prepare for, respond to, and control COVID-19 now should pay off for future shocks and create more sustainable, inclusive and responsive health systems in the future.^14^ They are also likely to have long-term implications that could change the system’s context. A challenge with the pandemic has been the need to act quickly to a rapidly changing situation, making outcomes difficult to predict and leaving little time to assess possible consequences.^26^ In addition, some of the proposed health system strategies and reforms for COVID, like scaling up telehealth, require long-term management changes and redesign of functions and structures^12^; consequences may not become apparent until later. The challenge will be to continue monitoring the system to observe consequences across all system elements, and to understand which absorptive, adaptive, and transformative changes have had a net positive effect.

 Despite keen awareness of the risks that COVID-19 posed to all of society, our understanding of how to monitor risks from other sectors is limited. Resilient systems should be proactively aware of risks as they develop and understand their potential impacts on the system, including risks that develop after the initial onset of a shock or that occur because of the shock. It is likely that more effective coordination and collaboration across sectors to monitor risks will be required. Analyzing information flows would be one way to identify information silos and describe bottlenecks in information-sharing interactions between sectors.^102^ One Health approaches to managing risk during pandemics and other shocks present an opportunity to learn how to incorporate risk awareness beyond the health system into resilience work, thanks to their inherently intersectoral model for integrating and sharing knowledge.^103^

 The results suggest that knowledge on health system resilience are often projected onto the whole health system, without considering of the resilience of different subsystems. What impact would the failure of the regional level of the health system have? What are the implications if the public health services subsystem has less capacity for resilience than the healthcare services? Future research on health system resilience will need to understand how the scales and subsystems support the resilience of the others.

 The importance of equity and fairness in the way that health systems react to crises has been a key lesson from the pandemic, particularly in regards to the social and economic consequences. This goes beyond the equitable and fair performance of the health system during a shock and links to our findings on the relationship between health system resilience and societal resilience. If health systems are expected to enact and sustain universal health coverage, then we should consider how to connect societal resilience to health system resilience. For instance, defining a successful COVID-19 response by metrics beyond the number of cases and deaths might help elucidate health system reforms that can improve societal resilience to future shocks. Expanding social protection schemes for health, like paid sick leave, and investing in social development and gender equality while still prioritizing health might create stronger, more equitable health systems that are better prepared for future shocks.^9,11,40^

 Although the COVID-19 literature was viewed through a single framework, Blanchet et al’s,^5^ the concepts in Kruk and colleagues’ Resilient Health System framework^104^ support the relevance of our findings. The Blanchet and Kruk frameworks both draw on ideas from systems thinking and complexity, seen in the capacities of the Dimensions framework and in the characteristics of awareness, self-regulation, integration, and adaptation in the Resilient Health System framework. In Kruk’s framework, rapid and open knowledge sharing among decision-makers and with communities creates system awareness; regional and global actors can be used to build capacity and improve self-regulation; collaborating and engaging with non-health sectors and the community builds trust and leads to integration; and adaptation is derived from resource flexibility, local and rapid decision making, and learning from experience. In the context of the two frameworks, the resilience aspects that were missing from the COVID-19 literature become more pressing. For instance, both frameworks support strong vertical integration inside the health system, yet the idea was relatively overlooked in the COVID-19 literature. It is unlikely that the idea is irrelevant to the shock caused by the COVID-19 pandemic. Does the absence of vertical integration in the literature indicate that it is relatively less important than other aspects of resilience, or that vertical integration is not being practiced during COVID-19? COVID-19 should serve as an impulse to reevaluate the relevance of resilience frameworks and promote the aspects that remain underexplored and underreported.

 We have identified lessons from the discussions on COVID-19 that show the limits of the framework and point to a wider role for resilient health systems in protecting health and in society. The Dimensions of Resilience Governance framework has clear utility in examining aspects of resilience, as seen by the variety of ideas in the COVID-19 discussions that are present in the framework. The coherence of the findings suggests that health system resilience is beneficial for understanding how to strengthen health systems for shocks. Our analysis presents a review of resilience through one lens. It would be worthwhile to conduct further analyses using other frameworks, to ascertain areas of overlap between concepts and to generate a more comprehensive assessment of where the concept of health system resilience currently stands.

## Ethical issues

 Not applicable.

## Competing interests

 Authors declare that they have no competing interests.

## Authors’ contributions

 DDS, FT, and KB conceptualized the study. DDS conducted the searches, data extraction and analysis. AD and SOK contributed to screening, data extraction, and analysis of the second search. All authors contributed to the final analysis. DDS drafted the first manuscript and all authors critically revised the manuscript.

## Funding

 The work was supported by the initiative “Foster Inter-University Initiatives and Collaborations” from the Swiss School of Public Health.

## Supplementary files

Supplementary file 1. Scientific Database Search Strategy for COVID-19 and Health System Resilience Literature.
