# Supplementary material for: Re-evaluating Our Knowledge of Health System Resilience During COVID-19: Lessons From the First Two Years of the Pandemic
Source: Int J Health Policy Manag. 2022 Dec 6;12:6659. doi: 10.34172/ijhpm.2022.6659 (PMC10125099; doi:10.34172/ijhpm.2022.6659)
Supplement: Supplementary file 1 — Scientific Database Search Strategy for COVID-19 and Health System Resilience Literature. [file ijhpm-12-6659-s001.pdf]

Article title: Re-evaluating Our Knowledge of Health System Resilience During COVID-19: Lessons From the First Two Years of the Pandemic

Journal name: International Journal of Health Policy and Management (IJHPM)

Authors' information: Dell D. Saulnier[1,2]*, Anna Duchenko[3], Sierra Ottilie-Kovelman[3], Fabrizio Tediosi[3,4], Karl Blanchet[5]

[1]Department of Clinical Sciences Malmö, Lund University, Malmö, Sweden.

[2]Department of Global Public Health, Karolinska Institutet, Stockholm, Sweden.

[3]Swiss Tropical and Public Health Institute, Basel, Switzerland.

[4]University of Basel, Basel, Switzerland.

[5]Geneva Centre of Humanitarian Studies, Faculty of Medicine, University of Geneva, Geneva, Switzerland.

(*Corresponding author: dell.saulnier@med.lu.se)

Supplementary file 1. Scientific Database Search Strategy for COVID-19 and Health System Resilience Literature

Medline

1. Exp Health Services/

2. Exp Delivery of Health Care/

3. Exp Health Services Research/

4. Exp Secondary Care/

5. 1 OR 2 OR 3 OR 4

6. health system.mp OR health systems.mp OR healthcare system.mp OR healthcare systems.mp OR health care system.mp OR health care systems.mp OR healthcare.mp OR health care.mp OR healthcare sector.mp OR healthcare sectors.mp OR health care sector.mp OR health care sectors.mp OR health service.mp OR health services.mp OR service delivery.mp OR healthcare service.mp OR healthcare services.mp OR health care service.mp OR health care services.mp

7. 5 OR 6

8. Resilience.mp OR resilient.mp OR resilie*.mp

9. 7 AND 8

10. covid-19.mp OR sars-cov-2.mp OR corona virus.mp OR coronavirus.mp

11. 9 AND 10

12. exp Mental Health/

13. exp Psychology/

14. exp Psychiatry/

15. exp Behavioral Symptoms/

16. 12 OR 13 OR 14 OR 15

17. 11 NOT 16

18. limit 17 to (human and English language and yr="2019 -Current")

Web of Science

1. TS=(“health system” OR “health systems” OR “healthcare system” OR “healthcare systems” OR “health care system” OR “health care systems” OR “healthcare” OR “health care” OR “healthcare sector” OR “healthcare sectors” OR “health care sector” OR “health care sectors” OR “health service” OR “health services” OR “service delivery” OR “healthcare service” OR “healthcare services” OR “health care service” OR “health care services”)

2. TS=(resilience OR resilient OR resilie*) OR AK=(resilience OR resilient OR resilie*) OR KP=(resilience OR resilient OR resilie*)

3. TS=(covid-19 OR sars-cov-2 OR “corona virus” OR coronavirus) OR AK=(covid-19 OR sars-cov-2 OR “corona virus” OR coronavirus) OR KP=(covid-19 OR sars-cov-2 OR “corona virus” OR coronavirus)

4. #1 AND #2 AND #3

5. TS=(“mental health” OR psychology OR psychiatry)

6. #4 NOT #5

7. #6 AND LANGUAGE: (English) Timespan=2019-2020

CINAHL

1. MH Health Services

2. MH Health Services Research

3. S1 OR S2

4. “health system” OR “health systems” OR “healthcare system” OR “healthcare systems” OR “health care system” OR “health care systems” OR “healthcare” OR “health care” OR “healthcare sector” OR healthcare sectors” OR “health care sector” OR “health care sectors” OR “health service” OR “health services” OR “service delivery” OR “healthcare service” OR “healthcare services” OR “health care service” OR “health care services”

5. S3 OR S4

6. Resilience OR resilient OR resilie*

7. S5 AND S6

8. covid-19 OR sars-cov-2 OR “corona virus” OR coronavirus

9. S7 AND S8

10. MH Mental Health

11. MH Psychology

12. MH Psychiatry

13. S9 NOT (S10 OR S11 OR S12)
